# Low bone mineral density is a potential risk factor for symptom onset and related with hypocitraturia in urolithiasis patients: a single-center retrospective cohort study

**DOI:** 10.1186/s12894-020-00749-5

**Published:** 2020-10-29

**Authors:** Kazumi Taguchi, Shuzo Hamamoto, Atsushi Okada, Yutaro Tanaka, Teruaki Sugino, Rei Unno, Taiki Kato, Ryosuke Ando, Keiichi Tozawa, Takahiro Yasui

**Affiliations:** Department of Nephro-urology, Nagoya City University Graduate School of Medical Sciences, Kawasumi 1, Mizuho-cho, Mizuho-ku, Nagoya, 4678601 Japan

**Keywords:** Urolithiasis, 24-h urine, Bone mineral density, T-score, Hypocitraturia

## Abstract

**Background:**

Patients with urolithiasis have a lower bone mineral density (BMD) than those without stones, suggesting a potential correlation between calcium stone formation and bone resorption disorders, including osteopenia and osteoporosis.

**Methods:**

To investigate the influence of BMD on clinical outcomes in urolithiasis, we performed a single-center retrospective cohort study to analyze patients with urolithiasis who underwent both BMD examination and 24-h urine collection between 2006 and 2015. Data from the national cross-sectional surveillance of the Japanese Society on Urolithiasis Research in 2015 were utilized, and additional data related to urinary tract stones were obtained from medical records. The primary outcome was the development of stone-related symptoms and recurrences during follow-up. A total of 370 patients were included in this 10-year study period.

**Results:**

Half of the patients had recurrent stones, and the two-thirds were symptomatic stone formers. While only 9% of patients had hypercalciuria, 27% and 55% had hyperoxaluria and hypocitraturia, respectively. There was a positive correlation between T-scores and urinary citrate excretion. Both univariate and multivariate analyses demonstrated that female sex was associated with recurrences (odds ratio = 0.44, *p* = 0.007), whereas a T-score < − 2.5 and hyperoxaluria were associated with symptoms (odds ratio = 2.59, *p* = 0.037; odds ratio = 0.45, *p* = 0.01; respectively).

**Conclusion:**

These results revealed that low T-scores might cause symptoms in patients with urolithiasis, suggesting the importance of BMD examination for high-risk Japanese patients with urolithiasis having hypocitraturia.

## Background

The prevalence of urolithiasis is increasing worldwide [1], with a reported recurrence rate of 15 per 100 person-years [2]. With the recognized relationship between urolithiasis and metabolic syndrome, the increasing prevalence of metabolic syndrome, such as obesity and diabetes mellitus, is also speculated to increase the prevalence of urolithiasis [3]. Urolithiasis is not a direct life-threatening disease, but a recent study indicated that it could indirectly cause death with its slightly increasing trend [4]. Therefore, prevention is essential in reducing its economic and medical burden; however, there are few useful biomarkers for monitoring and predicting disease severity and recurrence except for 24-h urine parameters.

Calcium-containing stones are the most prevalent, and this disease pathology is clearly based on the mineralization process [5]. Patients with urolithiasis, especially those whose stones are composed of calcium oxalate and phosphate, are reported to have abnormal mineral laboratory findings, such as hypercalcemia and hypercalciuria, as well as bone metabolic symptoms including fractures [6]. The bone mineral density (BMD) of patients with urolithiasis is lower than that of those without stones [7]; even the male adolescent population demonstrates similar findings [8]. In particular, osteoporotic states often cause hypercalciuria in patients with urolithiasis due to deterioration in bone resorption [9]. This evidence indicates that the pathogenesis of urolithiasis is linked to osteogenesis via mineral metabolism.

Worldwide guidelines [10–12] recommend 24-h urine collection for patients considered high-risk stone formers, such as those with staghorn stones, recurrences, and comorbidities like metabolic syndromes. However, the examination of osteogenesis parameters, including BMD, is usually not recommended by experts despite the overlap between urolithiasis and osteogenesis. In contrast, with 24-h urine collection, few urologists examine BMD in patients with urolithiasis; hence, the clinical importance of monitoring BMD in patients with urolithiasis has not been fully understood.

We previously investigated the potential therapeutic influence of bisphosphonates on postmenopausal women with urolithiasis, who showed improved BMD and reduced risk of calcium phosphate stone formation [13]. Since then, we have been monitoring BMD in patients with urolithiasis to better understand its role in the pathogenesis of urolithiasis. This study aimed to evaluate the association between BMD and clinical outcomes in urolithiasis, including not only mineral parameters such as 24-h urine collection but also symptoms and incidence of recurrences.

## Methods

### Study design and patient selection

**Fig. 1:**
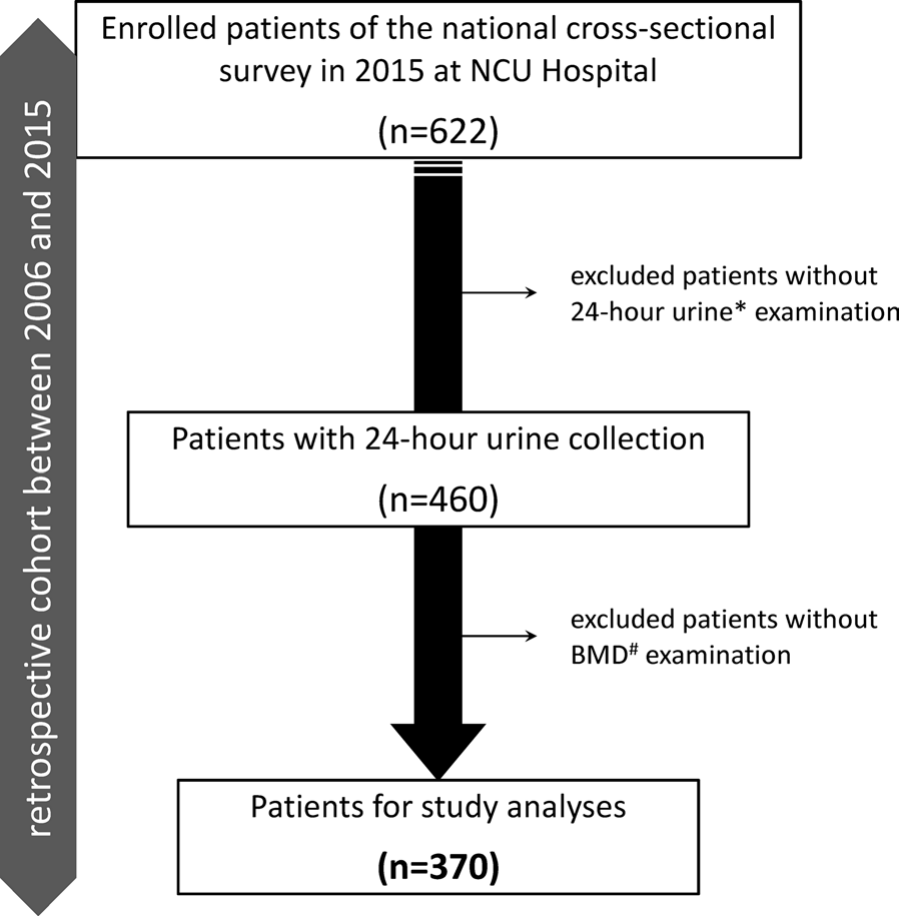
Study design chart. *Examined for high-risk stone formers using at least one collection after surgical intervention and/or during clinic follow-up. ^#^Examined for high-risk stone formers with suspicion of bone mineral abnormalities within 6 months of 24-h urine collection. NCU, Nagoya City University Hospital; BMD, bone mineral density

This retrospective cohort study was conducted at Nagoya City University (NCU) Hospital, a high-volume center. The institutional review board (IRB) approval was obtained from the medical research review board at NCU Graduate School of Medical Sciences (#60-19-0044), as well as Chiba University Graduate School of Medicine (#1962), Kanazawa Medical University (#226), and Osaka City University (#970) as national cross-sectional surveillance. The patients provided written informed consent. At the time of the national cross-sectional survey by the Japanese Society on Urolithiasis Research in 2015, all our patients with urinary stone disease diagnosed by radiographic evidence were included, as previously reported [14]. We utilized this surveillance data at NCU Hospital and selected patients with both 24-h urine collection and BMD examination at least once between 2006 and 2015 (Fig. 1). Patients younger than 18 years and those who had severe conditions due to other diseases were excluded from the study.

The primary outcome was the presence of symptoms during the follow-up and any recurrence, and the secondary outcome was the association between BMD data and 24-h urine parameters. The stone-related symptoms were confirmed by patients’ self-reports of pain, discomfort, hematuria, and/or stone passage, which seemed to be related to urolithiasis; recurrence was confirmed by past medical history with either patients’ self-reports or image findings.

### Data collection

Patient characteristics, stone-related symptoms, treatment history of urolithiasis, comorbidities, and BMD examination results, blood tests, and 24-h urine collection were obtained from the survey data and institutional electronic medical records. We also captured doctor’s lifestyle recommendations for fluid (> 2.5 L/day) and nutrition (increasing calcium-rich food and vegetables/fruits and reducing sodium and animal protein intake), which were obtained from the electronic medical records.

BMD was measured from the lumbar vertebra (L2-4) using dual-energy X-ray absorptiometry (DEXA) DELPHI A™; Hologic, Inc., Marlborough, MA, USA). With standard deviation (SD) based on the average BMD of young adults, the T-score was utilized for standard evaluation. The T-scores were categorized as normal (> − 1.0), osteopenia (between − 1.0 and − 2.5), and osteoporosis (< − 2.5), according to the World Health Organization guidelines.

### Statistical analysis

We utilized means ± SD and medians (25% and 75% interquartile range) for normally and non-normally distributed variables, respectively. Differences were identified using either Two-sample *t* test, Mann–Whitney *U* tests, or Fisher’s exact test. Differences were considered statistically significant at *α* < 0.05 with analyses using EZR (R Project, Vienna, Austria) [15].

## Results

Among 622 patients with urolithiasis at our institution from the national cross-sectional survey year in 2015, 370 were included in this 10-year study period. The background and sex comparison of the enrolled patients are summarized in Table 1. The sex comparison revealed that female patients were significantly younger at both disease onset and clinic visits and had lower BMI, lower recurrence rates, lower BMD and T-score/Z-scores, higher serum phosphate and urinary pH, and lower urinary phosphate, sodium, and oxalate excretion than male patients.

**Table 1:** Patients’ background and sex comparison of study population

	Total (n = 370)	Male (n = 233)	Female (n = 137)	*p *value
Age (years)	57.5 ± 14.6	55.5 ± 14.7	60.9 ± 13.9	0.001
BMI (kg/m^2^)	24.4 ± 4.9	25.2 ± 4.7	23.2 ± 4.8	0.002
Age of onset (years)	46.5 ± 16.7	44.5 ± 16.1	50.1 ± 17.5	0.01
Previous history of urolithiasis	174 (53.7)	122 (58.9)	52 (44.4)	0.02
Presence of symptoms at clinic visits	216 (62.1)	142 (64.3)	74 (58.3)	0.30
Family history of stone	26 (22.2)	16 (19.3)	10 (29.4)	0.23
DM	50 (14.1)	32 (14.3)	18 (13.8)	1.00
HLP	94 (26.6)	63 (28.3)	31 (23.8)	0.39
HTN	109 (30.7)	65 (29.0)	44 (33.6)	0.40
PHP	11 (3.3)	4 (1.9)	7 (5.6)	0.11
RTA	3 (0.9)	1 (0.5)	2 (1.6)	0.56
Fluid recommendation	295 (82.9)	193 (85.8)	102 (77.9)	0.12
Nutrition recommendation	332 (93.3)	211 (93.8)	121 (92.4)	0.84
Thiazide use	1 (0.3)	0 (0.0)	1 (0.8)	0.37
VitD supplementation	4 (1.2)	0 (0.0)	4 (3.1)	0.02
BMD (g/cm^2^)	0.91 ± 0.18	0.95 ± 0.17	0.84 ± 0.18	< 0.001
T-score	− 1.00 ± 1.48	− 0.58 ± 1.13	− 1.71 ± 1.72	< 0.001
Z-score	0.40 ± 1.48	0.12 ± 1.23	0.86 ± 1.73	< 0.001
Serum Ca (mg/dL)	9.3 ± 0.4	9.3 ± 0.4	9.3 ± 0.5	0.44
Serum P (mg/dL)	3.2 ± 0.5	3.1 ± 0.5	3.5 ± 0.4	< 0.001
Serum PTH (mg/dL)	46.7 ± 22.9	44.5 ± 21.8	49.6 ± 24.2	0.16
Urinary pH	6.50 [6.00, 7.00]	6.50 [6.00, 7.00]	6.75 [6.25, 7.25]	0.01
Urinary volume (L/day)	1.45 [1.05, 2.00]	1.50 [1.10, 2.05]	1.35 [1.00, 1.95]	0.05
Hypercalciuria	32 (8.6)	23 (9.9)	9 (6.6)	0.34
Hyperphosphaturia	35 (9.5)	30 (12.9)	5 (3.6)	0.003
Hypernatriuria	1 (0.3)	0 (0.0)	1 (0.7)	0.37
Hyperoxaluria	98 (26.5)	71 (30.5)	27 (19.7)	0.03
Hypocitraturia	203 (54.9)	124 (53.2)	79 (57.7)	0.45
Urinary Ca (g/day)	0.14 [0.09, 0.20]	0.14 [0.09, 0.21]	0.15 [0.09, 0.20]	0.97
Urinary P (g/day)	0.66 [0.50, 0.83]	0.71 [0.54, 0.87]	0.58 [0.41, 0.73]	< 0.001
Urinary Na (g/day)	2.54 [1.84, 3.31]	2.85 [1.98, 3.51]	2.13 [1.65, 2.96]	< 0.001
Urinary Ox (mg/day)	27.7 [20.6, 37.2]	29.7 [22.8, 40.8]	23.5 [16.9, 32.4]	< 0.001
Urinary Cit (mg/day)	347 [209, 508]	362 [216, 512]	329 [204, 508]	0.53

Values are presented as mean ± SD, n (%), or medians [interquartile range]

Definitions of urine abnormalities: hypercalciuria ≥ 300 mg/day; hyperphosphaturia ≥ 3 g/day; hypernatriuria ≥ 5.8 g/day; hyperoxaluria ≥ 40 mg/day; hypocitraturia ≤ 320 mg/day

BMI, body mass index; DM, diabetes mellitus, HLP, hyperlipidemia; HTN, hypertension, PHP, pseudohypoparathyroidism; RTA, renal tubular acidosis; VitD, vitamin D; BMD, bone mineral density, SD, standard deviation, Ca, calcium, P, phosphorus, PTH, parathyroid hormone; Na, sodium; Ox, oxalate; Cit, citrate

Among female patients, 77% were postmenopausal women, were significantly older, had a larger population with hypocitraturia and hypertension as comorbidities, and had much lower BMD and T-scores than premenopausal women (Table 2). The other background characteristics affecting bone and/or calcium metabolism, including nutrition recommendation, bisphosphonate/sodium potassium citrate/thiazide usage, and vitamin D supplementation, did not differ between premenopausal and postmenopausal women.

**Table 2:** Background differences between premenopausal and postmenopausal women with urolithiasis

	Premenopausal female (n = 30)	Postmenopausal female (n = 103)	*p *value
Age (years)	41.1 ± 7.7	70.0 ± 9.2	< 0.001
BMI (kg/m^2^)	23.1 ± 4.6	23.4 ± 4.9	0.77
Age of onset (years)	33.2 ± 9.0	56.4 ± 16.2	< 0.001
Previous history of urolithiasis	14 (46.7)	38 (44.7)	0.67
Presence of symptoms at clinic visits	15 (50.0)	55 (58.5)	0.53
Family history of stone	5 (16.7)	5 (21.7)	0.22
DM	2 (6.7)	16 (16.7)	0.24
HLP	3 (10.0)	28 (29.2)	0.05
HTN	4 (13.3)	40 (41.2)	0.01
PHP	2 (6.9)	5 (5.3)	0.67
RTA	0 (0.0)	2 (2.1)	1.00
Fluid recommendation	24 (80.0)	74 (76.3)	0.85
Nutrition recommendation	28 (93.3)	89 (91.8)	1.00
Bisphosphonate use	2 (6.7)	14 (13.6)	0.52
NaKCit use	10 (33.3)	27 (28.4)	0.65
Thiazide use	0 (0.0)	1 (1.1)	1.00
VitD supplementation	1 (3.3)	3 (3.2)	1.00
BMD (g/cm^2^)	0.93 ± 0.15	0.81 ± 0.18	0.001
T-score	− 0.83 ± 1.47	− 2.01 ± 1.72	0.001
Serum Ca (mg/dL)	9.1 ± 0.5	9.3 ± 0.4	0.05
Serum P (mg/dL)	3.5 ± 0.5	3.5 ± 0.4	0.84
Serum PTH (mg/dL)	44.7 ± 16.5	51.8 ± 26.4	0.28
Urinary pH	7.00 [6.50, 7.25]	6.75 [6.38, 7.12]	0.56
Urinary volume (L/day)	1.25 [0.96, 1.64]	1.40 [1.05, 1.95]	0.36
Hypercalciuria	0 (0.0)	9 (8.7)	0.21
Hyperphosphaturia	2 (6.7)	3 (2.9)	0.32
Hypernatriuria	1 (3.4)	0 (0.0)	0.29
Hyperoxaluria	6 (20.0)	20 (19.4)	1.00
Hypocitraturia	11 (36.7)	66 (64.1)	0.01
Urinary Ca (g/day)	0.12 [0.08, 0.19]	0.15 [0.09, 0.22]	0.19
Urinary P (g/day)	0.60 [0.41, 0.76]	0.56 [0.40, 0.73]	0.44
Urinary Na (g/day)	2.05 [1.52, 3.01]	2.17 [1.66, 2.92]	0.96
Urinary Ox (mg/day)	24.9 [21.0, 34.3]	23.3 [16.2, 29.1]	0.21
Urinary Cit (mg/day)	395 [242, 548]	296 [178, 458]	0.07

Values are presented as mean ± SD, n (%), or medians [interquartile range]

BMI, body mass index; DM, diabetes mellitus, HLP, hyperlipidemia; HTN, hypertension, PHP, pseudohypoparathyroidism; RTA, renal tubular acidosis; NaKCit, sodium potassium citrate; VitD, vitamin D; BMD, bone mineral density, SD, standard deviation, Ca, calcium, P, phosphorus, PTH, parathyroid hormone; Na, sodium; Ox, oxalate; Cit, citrate

Additional univariate analysis comparing T-score ranges demonstrated that among patients with urolithiasis, those who had low T-scores were older and had lower BMI, higher prevalence of pseudohypoparathyroidism and renal tubular acidosis, higher bisphosphonate and thiazide use, and a lower and higher probability of having hyperphosphaturia and hypocitraturia, respectively (Table 3).

**Table 3:** Association between bone mineral density, disease severity, and osteogenesis parameters in patients with urolithiasis

	T-score			*p* value
	> − 1.0 (n = 186)	− 1 to − 2.5 (n = 135)	< − 2.5 (n = 49)	
Age (years)	55.3 ± 14.6	57.8 ± 14.6	64.9 ± 12.4	< 0.001
BMI (kg/m^2^)	25.8 ± 5.1	23.5 ± 4.5	21.6 ± 2.5	< 0.001
Age of onset (years)	44.9 ± 16.0	46.1 ± 17.1	54.3 ± 17.3	0.01
Recurrence	89 (54.3)	65 (56.0)	20 (45.5)	0.48
Previous history of urolithiasis	113 (63.8)	69 (56.1)	34 (70.8)	0.16
Presence of symptoms during clinic visits	9.3 ± 0.4	9.3 ± 0.4	9.3 ± 0.5	0.39
Family history of stones	13 (19.4)	12 (32.4)	1 (7.7)	0.13
DM	24 (13.3)	19 (15.0)	7 (14.9)	0.91
HLP	51 (28.5)	32 (25.2)	11 (23.4)	0.70
HTN	56 (31.1)	36 (28.3)	17 (35.4)	0.66
PHP	2 (1.2)	4 (3.2)	5 (11.1)	0.004
RTA	1 (0.6)	0 (0.0)	2 (4.4)	0.02
Fluid recommendation	149 (82.3)	105 (82.7)	41 (85.4)	0.54
Nutrition recommendation	169 (93.4)	119 (93.7)	44 (91.7)	0.66
Bisphosphonate use	1 (0.5)	6 (4.4)	15 (30.6)	< 0.001
NaKCit use	37 (20.7)	31 (24.4)	12 (26.1)	0.63
Thiazide use	0 (0.0)	0 (0.0)	1 (2.2)	0.04
VitD supplementation	2 (1.2)	0 (0.0)	2 (4.3)	0.07
Serum Ca (mg/dL)	9.3 ± 0.4	9.3 ± 0.4	9.3 ± 0.5	0.39
Serum P (mg/dL)	3.2 ± 0.5	3.2 ± 0.6	3.4 ± 0.4	0.33
Serum PTH (mg/dL)	44.5 ± 21.4	47.2 ± 23.8	56.4 ± 26.3	0.21
Urinary pH	6.50 [6.00, 7.00]	6.58 [6.00, 7.00]	6.75 [6.12, 7.00]	0.47
Urinary volume (L/day)	1.50 [1.03, 2.15]	1.40 [1.10, 1.95]	1.40 [1.13, 1.88]	0.72
Hypercalciuria	13 (7.0)	17 (12.6)	2 (4.1)	0.10
Hyperphosphaturia	23 (12.4)	12 (8.9)	0 (0.0)	0.03
Hyperoxaluria	56 (30.1)	32 (23.7)	10 (20.4)	0.26
Hypocitraturia	92 (49.5)	76 (56.3)	35 (71.4)	0.02

Values are presented as means ± SD, n (%), or medians [interquartile range]

Definitions of urine abnormalities: hypercalciuria ≥ 300 mg/day; hyperphosphaturia ≥ 3 g/day; hypernatriuria ≥ 5.8 g/day; hyperoxaluria ≥ 40 mg/day; hypocitraturia ≤ 320 mg/day

BMI, body mass index; DM, diabetes mellitus, HLP, hyperlipidemia; HTN, hypertension, PHP, pseudohypoparathyroidism; RTA, renal tubular acidosis; VitD, vitamin D; SD, standard deviation, Ca, calcium, P, phosphorus, PTH, parathyroid hormone

**Fig. 2:**
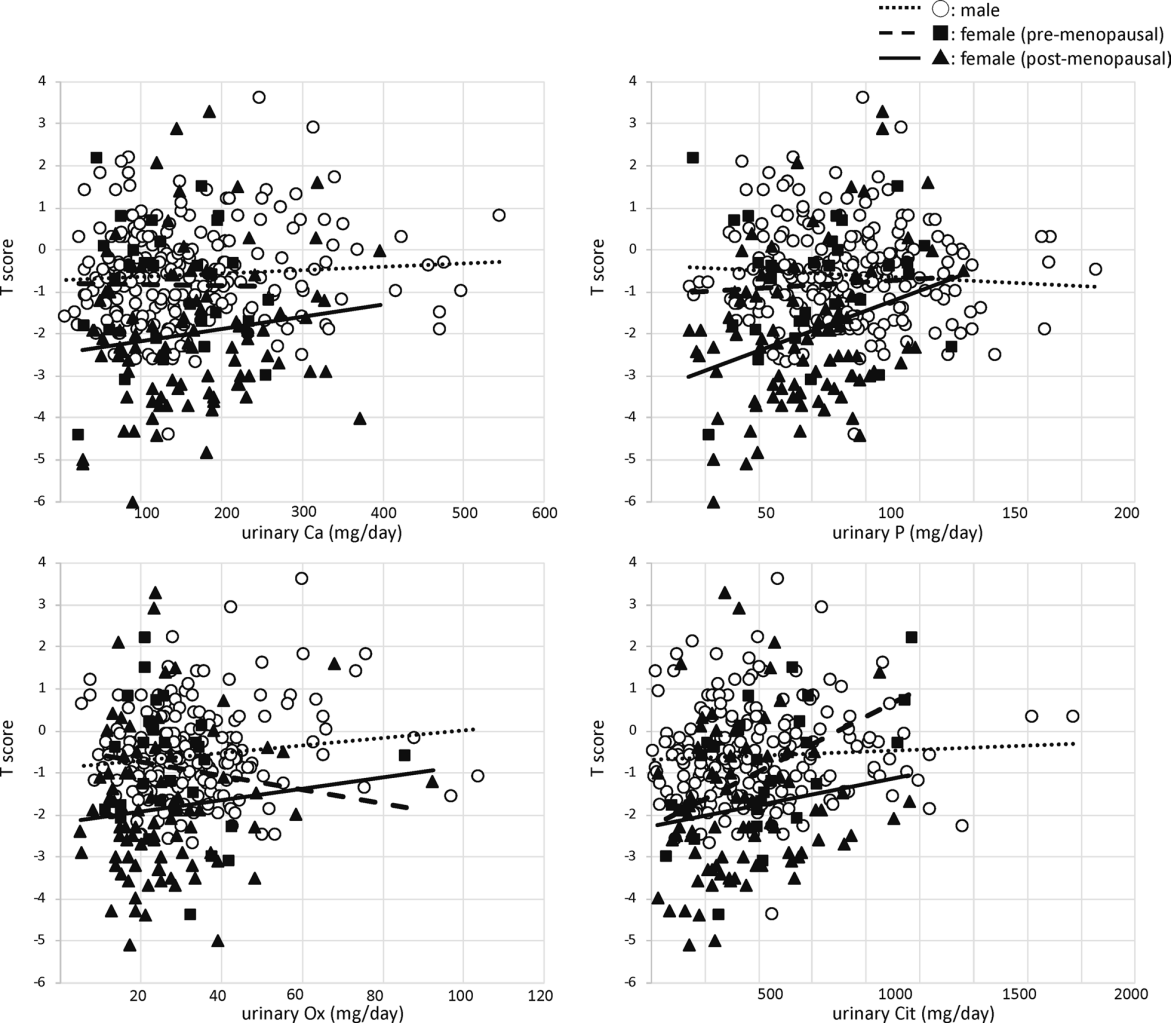
Scatterplots of the results of Spearman’s rank correlation tests between T-scores and urinary calcium (male: coefficient = 0.06, *p* = 0.40; premenopausal female: coefficient = − 0.03, *p* = 0.89; postmenopausal female: coefficient = 0.11, *p* = 0.41), phosphate (male: coefficient = 0.23, *p* < 0.001; premenopausal female: coefficient = 0.07, *p* = 0.70; postmenopausal female: coefficient = 0.32, *p* = 0.01), oxalate (male: coefficient = 0.14, *p* = 0.05; premenopausal female: coefficient = 0.21, *p* = 0.31; postmenopausal female: coefficient = 0.11, *p* = 0.44), and citrate excretion (male: coefficient = 0.18, *p* = 0.01; premenopausal female: coefficient = 0.57 *p* = 0.003; postmenopausal female: coefficient = 0.22, *p* = 0.12). Ca, calcium; P, phosphate; Ox, oxalate; Cit, citrate

Figure 2 shows the scatterplots of the correlation analyses between T-scores and urinary parameters from the 24-h urine collection. There were positive correlations between T-scores and urinary phosphate excretion in male and postmenopausal female patients and urinary citrate excretion in male and premenopausal female patients. No association was found between T-scores and urinary calcium and oxalate excretion.

We further performed logistic regression analyses to determine the potential risks associated with patients’ clinical outcomes with urolithiasis, such as the presence of symptoms during the follow-up period and the existence of stone recurrence before and during the follow-up period. Although the univariate analysis did not show any association between stone recurrence and BMD as well as urinary parameters, both univariate and multivariate analyses demonstrated that female sex was negatively associated with recurrences (odds ratio = 0.44, *p* = 0.007 in the multivariate analysis) (Table 4). In addition to recurrence, both univariate and multivariate analyses showed that hyperoxaluria was associated with a lower chance of developing symptoms during follow-up (odds ratio = 0.45, *p* = 0.01); however, a T-score < − 2.5 was associated with a higher possibility of developing symptoms (odds ratio = 2.59, *p* = 0.037) (Table 5).

**Table 4:** Logistic regression analyses of bone mineral density and urinary parameters for stone recurrence

Factors	Univariate analysis		Multivariate analysis	
	OR (95% CI)	*p* value	OR (95% CI)	*p* value
T-score < − 2.5	0.68 (0.36–1.29)	0.24	0.86 (0.36–2.05)	0.73
Hypercalciuria	2.05 (0.86–4.86)	0.1	3.12 (0.92–10.60)	0.068
Hyperoxaluria	1.38 (0.83–2.28)	0.22	0.92 (0.48–1.75)	0.8
Hyperphosphaturia	1.29 (0.62–2.71)	0.5	0.79 (0.26–2.43)	0.68
Hypocitraturia	0.97 (0.63–1.51)	0.91	1.01 (0.56–1.81)	0.98
Female sex	0.56 (0.35–0.88)	0.012	0.44 (0.24–0.80)	0.007
age	1.01 (0.99–1.03)	0.47	1.00 (0.98–1.02)	0.83
body mass index	1.00 (0.95–1.06)	0.99	0.98 (0.92–1.04)	0.46

Data were adjusted by age, body mass index, T-score, hypercalciuria, hyperoxaluria, hyperphosphaturia, hypocitraturia, and female sex

Definitions of urine abnormalities: hypercalciuria ≥ 300 mg/day; hyperphosphaturia ≥ 3 g/day; hypernatriuria ≥ 5.8 g/day; hyperoxaluria ≥ 40 mg/day; hypocitraturia ≤ 320 mg/day

**Table 5:** Logistic regression analyses of bone mineral density and urinary parameters for symptomatic stones

Factors	Univariate analysis		Multivariate analysis	
	OR (95% CI)	*p* value	OR (95% CI)	*p* value
T-score < − 2.5	1.57 (0.81–3.06)	0.18	2.59 (1.06–6.36)	0.037
Hypercalciuria	1.18 (0.53–2.61)	0.69	1.35 (0.49–3.71)	0.56
Hyperoxaluria	0.60 (0.37–0.97)	0.038	0.45 (0.24–0.83)	0.01
Hyperphosphaturia	0.99 (0.48–2.04)	0.97	1.20 (0.43–3.36)	0.72
Hypocitraturia	0.88 (0.57–1.36)	0.57	1.24 (0.72–2.13)	0.44
Female sex	0.78 (0.50–1.21)	0.27	0.82 (0.46–1.45)	0.49
age	0.99 (0.98–1.01)	0.25	1.00 (0.98–1.01)	0.63
body mass index	1.00 (0.95–1.05)	0.88	1.01 (0.96–1.07)	0.66

Data were adjusted by age, body mass index, T-score, hypercalciuria, hyperoxaluria, hyperphosphaturia, hypocitraturia, and female gender

Definitions of urine abnormalities: hypercalciuria ≥ 300 mg/day; hyperphosphaturia ≥ 3 g/day; hypernatriuria ≥ 5.8 g/day; hyperoxaluria ≥ 40 mg/day; hypocitraturia ≤ 320 mg/day

## Discussion

Urolithiasis is known for its high prevalence and recurrence rate; therefore, close follow-up is important for preventing stone relapse and symptomatic events for better patient care [1, 2] In this study, we tried to investigate the relationship between the BMD and follow-up outcomes of patients with urolithiasis in clinical practice. Patients at high risk of urolithiasis, such as those with metabolic syndrome and metabolic abnormalities [16] should be carefully evaluated and followed-up; we speculate that the screening of BMD could be useful for reduce urinary risk factors including hypocitraturia and future symptom onset.

The relationship between urolithiasis and BMD was first reported in 1976 by Alhava et al. [17]. In their cohort of 21 male and 54 female participants, they found that the BMD was statistically lower in patients with urolithiasis than in healthy controls of both sexes. Since then, the association of urolithiasis with hypercalciuria and low BMD, particularly in postmenopausal women, has been recognized [18]. The main concern regarding bone metabolism in patients with urolithiasis is not only about having a higher chance of recurrence but also having a potential risk for fractures. A retrospective cohort study in the United Kingdom demonstrated that urolithiasis was associated with higher fracture risk, especially in adolescent boys and older women [19]. Similarly, two large cohort studies in the United States revealed that nephrolithiasis was associated with a markedly high risk of wrist fractures in both men and women (relative risk: 1.20) [20]. Interestingly, the Women’s Health Initiative report indicated a significant association between urolithiasis and incidental total fractures in postmenopausal women by unadjusted analyses; however, covariate-adjusted analyses revealed no statistical association between them [21]. Although there is no absolute conclusion, a recent meta-analysis suggested that patients with nephrolithiasis had significantly lower T-scores, was four times more likely to have osteoporosis, and had a potentially increased risk of fractures [7].

The low BMD in patients with urolithiasis, especially calcium-containing stone formers, is caused by calcium metabolism disorders including hypercalciuria [6, 22]. In fact, patients with urolithiasis have a high occurrence rate of hypercalciuria, up to 50% reported in the literature [23]. Unlike the results of previous reports from Europe and the United States [9, 24], our cohort had only 8.6% hypercalciuria in both men and women but had a higher prevalence of hypocitraturia. Similar to our study cohort, a Japanese cohort also had a lower prevalence of hypercalciuria [18]. Low BMD is found to be associated with hypercalciuria; this could be an independent risk factor for developing urolithiasis [25–27]. Owing to our unique demography, a low prevalence of hypercalciuria was observed in those with low BMD and postmenopausal women; we hypothesize that a different mechanism from hypercalciuria may cause a lower BMD in Japanese patients with urolithiasis. This needs further essential investigation. Our study also demonstrated the demographic differences between premenopausal and postmenopausal women with urolithiasis. Despite the relatively low number of patients in our study, the finding that postmenopausal women had a higher prevalence of hypertension, lower BMD, and hypocitraturia was consistent with current evidence [28], implying that postmenopausal status was associated with a higher risk of urolithiasis.

The current study also demonstrated some correlation between BMD and urinary parameters, such as hyperphosphaturia and hypocitraturia. There was a positive association between T-scores and urinary phosphate excretion in men and postmenopausal women with urolithiasis. Although no prior research directly detected this relationship, a few papers indicated the relationship between low BMD and phosphaturia [29, 30]. Since the bone resorption mechanism involves phosphate metabolism, which is also regulated by the intestinal phosphate absorption, hyperphosphaturia in urolithiasis may be linked to lower BMD; this may explain the risk for stone development. Furthermore, we found that urinary citrate excretion was positively associated with T-scores in men and premenopausal women with urolithiasis. Citrate is considered to decrease with acidosis under circumstances of increasing bone resorption; therefore, patients with urolithiasis with osteopenia or osteoporosis tend to have hypocitraturia [24, 31]. In fact, some papers indicated that potassium citrate treatment reversed low BMD [32, 33]. Such evidence suggests that awareness of urinary citrate levels is essential for evaluating stone development risk factors, including BMD.

Most importantly, logistic regression analyses revealed that female sex was associated with a decreased odds ratio for stone recurrence, whereas osteoporosis status (T-score < − 2.5) was associated with an increased odds ratio for developing stone symptoms; however, hyperoxaluria was associated with a decreased odds ratio for developing stone symptoms. Interestingly, this result of association between T-scores < − 2.5 and symptomatic stones was not observed on univariate analysis; this was probably due to the presence of confounders such as age, BMI, sex, and presence of urinary abnormalities. Examining BMD in patients with urolithiasis is important for effective follow-up; therefore, in real-world practice, this BMD evaluation may be performed in patients with a high-risk of urolithiasis at initial metabolic evaluation.

This study has several limitations, the primary being the study design. Although we could capture a large number of follow-up patients with urolithiasis who had both BMD and 24-h urine examinations, this single-center retrospective cohort study may not reflect daily practice data around the world. As described above, the prevalence of hypercalciuria was quite low, unlike previously reported rates among other ethnicities, suggesting a potential difference in calcium metabolism between the Asian population and others. Additionally, there is a lack of evidence regarding the stone composition and dietary/fluid records, which affect data interpretation. Preventive measures, such as bisphosphonates and NaKCit, may be biased by the preferences of physicians who diagnose and record the appropriate disease codes for connecting with the national insurance system. Lastly, our study did not include some bone turnover markers [6, 9] such as osteocalcin, β-cross-laps, and 25-hydroxy vitamin D, useful for evaluating bone metabolism; however, we believe that the current data set represents real-world data more accurately than currently available data.

## Conclusion

Our cross-sectional study on 370 patients with urolithiasis undergoing BMD and 24-h urine examinations revealed that lower BMD represented as T-scores, was associated with hyperphosphaturia and hypocitraturia. Moreover, logistic regression analyses revealed that a lower T-score was associated with increased odds ratios for stone symptoms during follow-up. These novel findings suggest that examining BMD could be a useful tool for effective follow-up of urolithiasis; this may prevent future risks of stone development and may influence current practice strategy.

## Data Availability

All data generated or analyzed during this study are included in this published article and are available from the corresponding author on reasonable request.

## Abbreviations

BMD: Bone mineral density
BMI: Body mass index
DEXA: Dual-energy X-ray absorptiometry
NCU: Nagoya City University
SD: Standard deviation
